# Supplementary material for: A second view on the evolution of flight in stick and leaf insects (Phasmatodea)
Source: BMC Ecol Evol. 2022 May 12;22:62. doi: 10.1186/s12862-022-02018-5 (PMC9097326; doi:10.1186/s12862-022-02018-5)
Supplement: Supplementary file 1 — Additional file 1: Figure S1. Maximum Likelihood phylogeny based on the best-scoring tree with nodal support (UFBoot and SH-aLRT) at each node (Files S1 and S2). Lock symbols represent constrained clades (B1 constraints). Character states for wings and ocelli are depicted at tips for females and males. Figure S2. Ancestral state reconstruction for males. Analysis based on the BI tree with B1 constraints (see lock symbols at nodes; File S3). Nodal support values (< 1 posterior probability) depicted at each node. Stars represent the fossils used for calibration and numbering corresponds to Table S9. T, Timematodea; ASCH, Aschiphasmatidae; A, Agathemeridae; PSEU, Pseudophasmatidae; HN, Heteronemiinae; DIAPH, Diapheromerinae; HET, Heteropterygidae; GRA, Gratidiidae sensu Cliquennois [70]; CLI, Clitumninae sensu Cliquennois [70]; BAC, Bacillinae sensu Cliquennois [70]; PHA, Pharnaciinae + Prosentoria; LANCEO, Lanceocercata; X, Xenophasmina; ST, Stephanacridini; P, Palophidae; CLA, Cladomorphinae; LONCH, Lonchodinae, NEC, Necrosciinae; AFR/MAD, African/Malagasy group including Achriopteridae, Anisacanthidae, Antongiliidae sensu Cliquennois [70], Damasippoididae and Xylicinae sensu Cliquennois [70]; PHYLL, Phylliidae. Figure S3. Ancestral state reconstruction for males. Analysis based on the BI tree with B3 constraints (see lock symbols at nodes; File S5). Nodal support values (< 1 posterior probability) depicted at each node. Stars represent the fossils used for calibration and numbering corresponds to Table S9. Abbreviations as in caption of Fig. S2. Figure S4. Ancestral state reconstruction for males. Analysis based on the BI tree with B2 constraints (see lock symbols at nodes; File S4). Nodal support values (< 1 posterior probability) depicted at each node. Stars represent the fossils used for calibration and numbering corresponds to Table S9. Abbreviations as in caption of Fig. S2. Figure S5. Ancestral state reconstruction for females. Analysis based on the BI tree wi [file 12862_2022_2018_MOESM1_ESM.pdf]

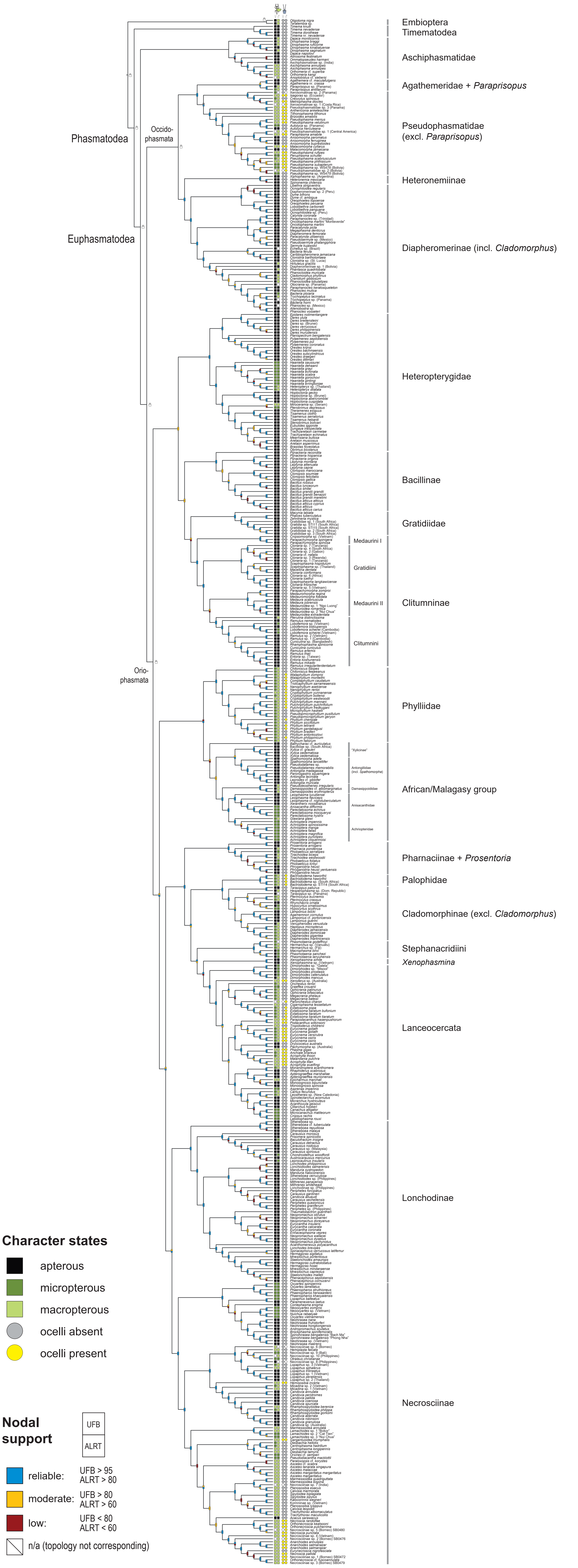

Figure S1. Maximum Likelihood phylogeny based on the best-scoring tree with nodal support (UFBoot and SH-aLRT) at each node (Files S1 and S2). Lock symbols represent constrained clades (B1 constraints). Character states for wings and ocelli are depicted at tips for females and males.

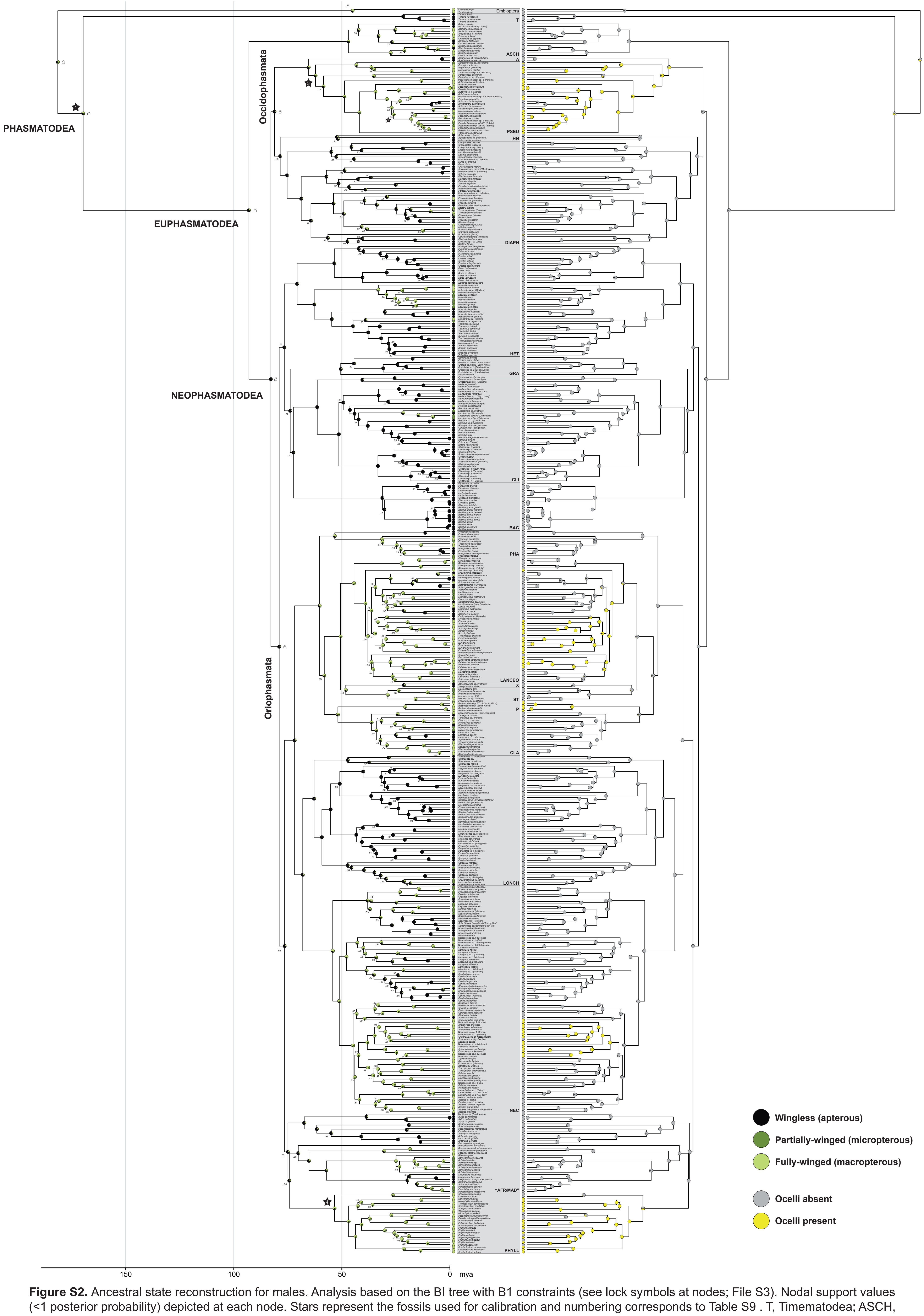

Figure S2. Ancestral state reconstruction for males. Analysis based on the BI tree with B1 constraints (see lock symbols at nodes; File S3). Nodal support values (<1 posterior probability) depicted at each node. Stars represent the fossils used for calibration and numbering corresponds to Table S9 . T, Timematodea; ASCH, Aschiphasmatidae; A, Agathemeridae; PSEU, Pseudophasmatidae; HN, Heteronemiinae; DIAPH, Diapheromerinae; HET, Heteropterygidae; GRA, Gratidiidae sensu Cliquennois [67]; CLI, Clitumninae sensu Cliquennois [67]; BAC, Bacillinae sensu Cliquennois [67]; PHA, Pharnaciinae + Prosentoria; ; LANCEO, Lanceo-cercata; X, Xenophasmina; ST, Stephanacridini; P, Palophidae; CLA, Cladomorphinae; LONCH, Lonchodinae, NEC, Necrosciinae; AFR/MAD, African/Malagasy group including Achriopteridae, Anisacanthidae, Antongiliidae sensu Cliquennois [67], Damasippoididae and Xylicinae sensu Cliquennois [67]; PHYLL, Phylliidae.

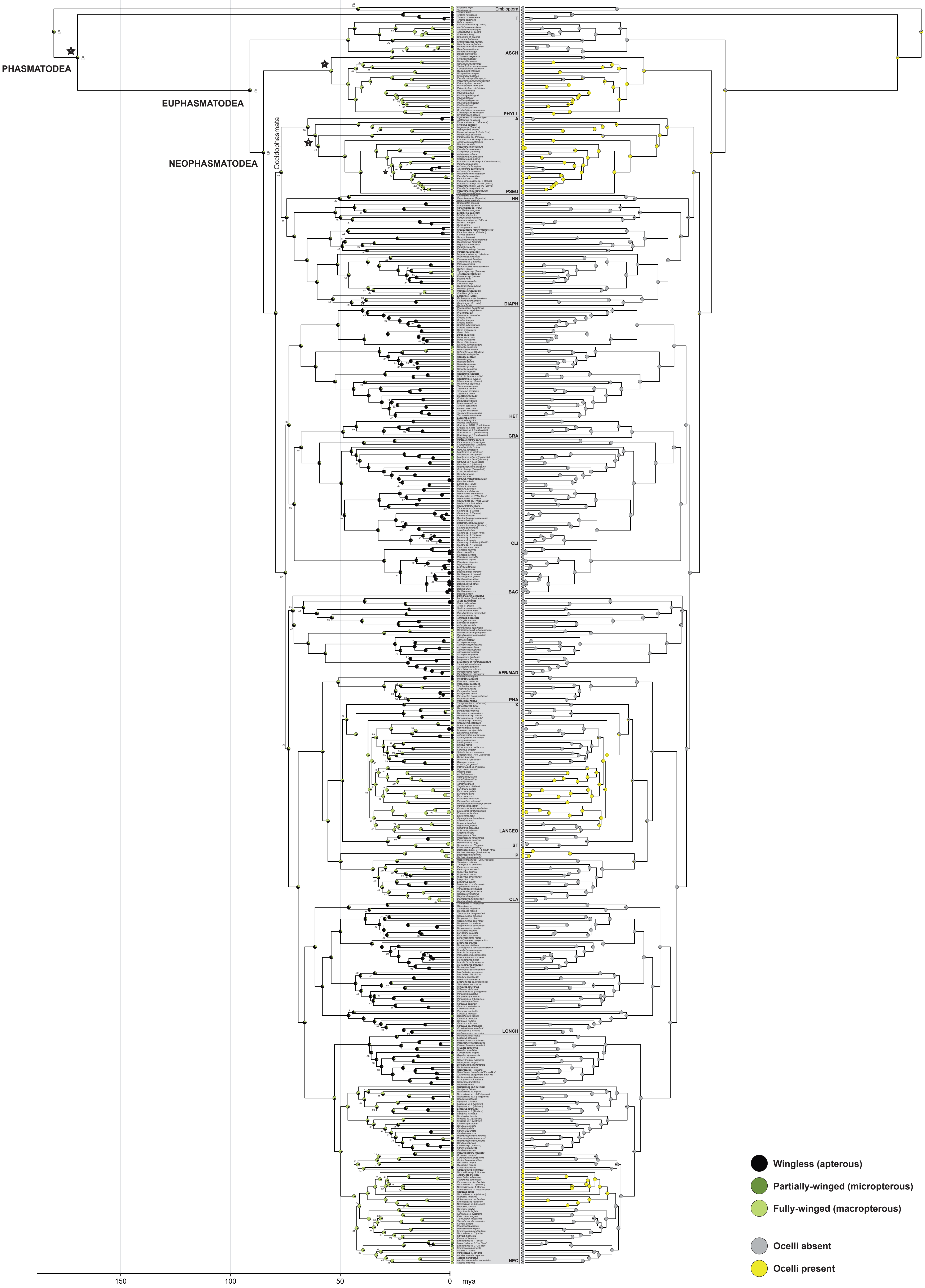

Figure S3. Ancestral state reconstruction for males. Analysis based on the BI tree with B3 constraints (see lock symbols at nodes; File S5). Nodal support values (<1 posterior probability) depicted at each node. Stars represent the fossils used for calibration and numbering corresponds to Table S9. Abbreviations as in caption of Figure S2.

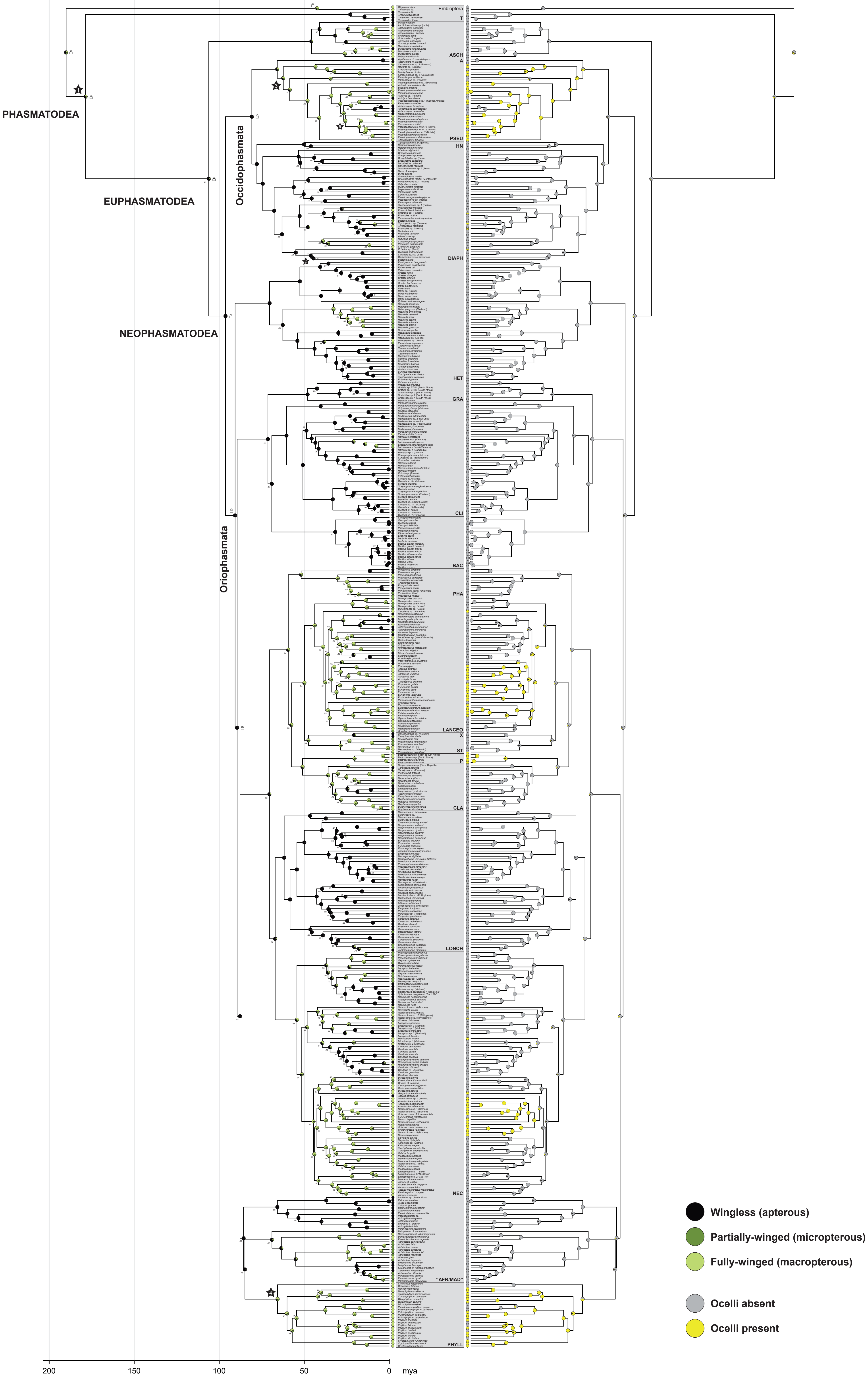

Figure S4. Ancestral state reconstruction for males. Analysis based on the BI tree with B2 constraints (see lock symbols at nodes; File S4). Nodal support values (<1 posterior probability) depicted at each node. Stars represent the fossils used for calibration and numbering corresponds to Table S9. Abbreviations as in caption of Figure S2.

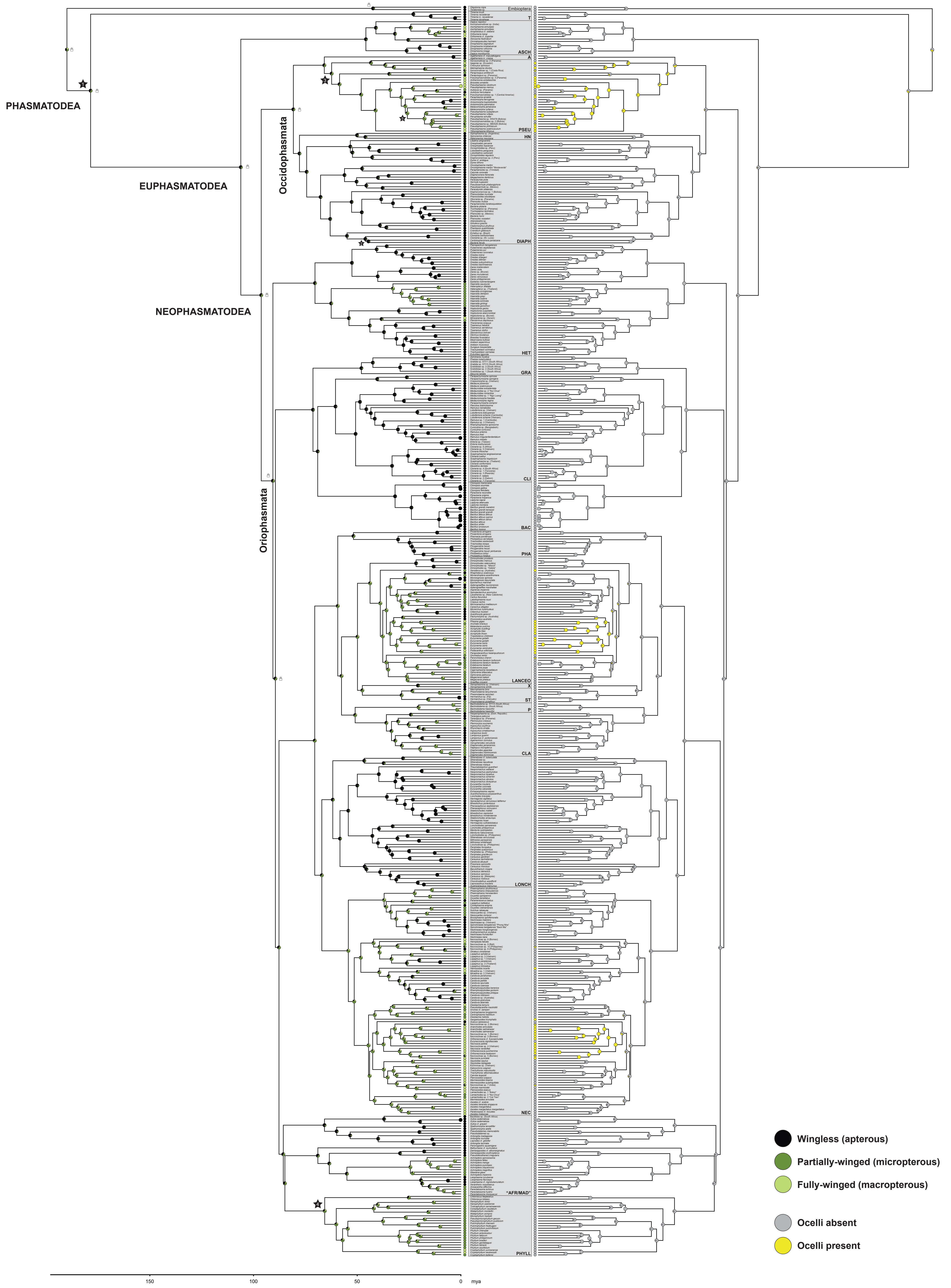

Figure S5. Ancestral state reconstruction for females. Analysis based on the BI tree with B2 constraints (see lock symbols at nodes; File S4). Nodal support values and divergence times are identical to those in Figure S4. Stars represent the fossils used for calibration and numbering corresponds to Table S9. Abbreviations as in caption of Figure S2.

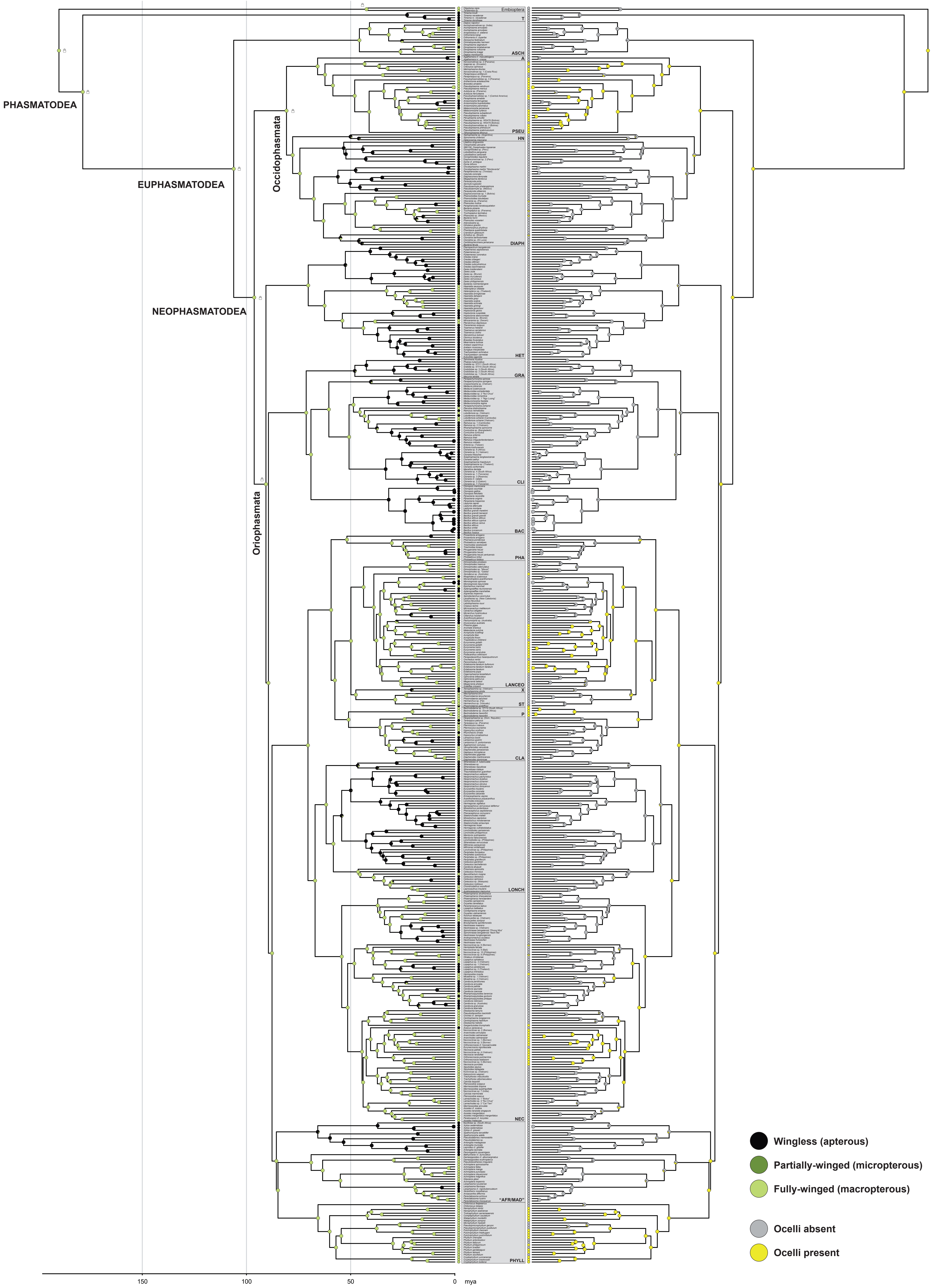

Figure S6. Ancestral state reconstruction for the binary dataset of males using the IRR model. Analysis based on the BI tree with B2 constraints (see lock symbols at nodes; File S4). Nodal support values and divergence times are identical to those in Figure S4. Stars represent the fossils used for calibration, and numbering corresponds to Table S9. Abbreviations as in caption of Figure S2.

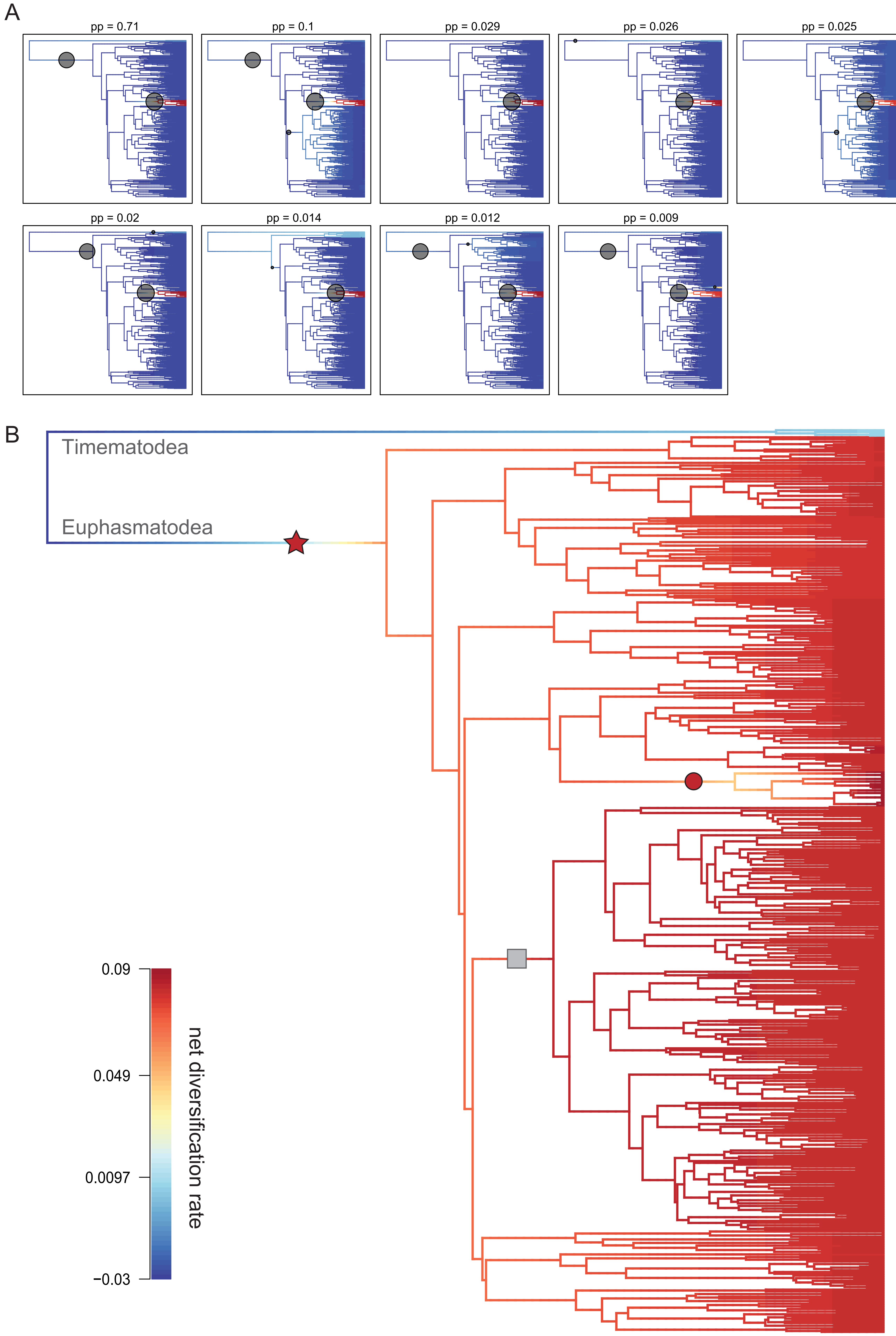

Figure S7. Phylorate plots resulting from diversification rate estimation in BAMM. (A) Credible set of shift configurations with posterior probabilities (pp). Mean rate parameters are model-averaged across all samples assignable to a given configuration. (B) Phylorate plot of net diversification. Model shifts are depicted as symbols on branches, with star (=Euphasmatodea) and circle (=European Bacillinae) according to the best shift configuration. The red colouration indicates rate acceleration. The grey square represents an additional potential rate shift added from the second best configuration (clade includes Pharnaciinae+ Prosentoria, Palophidae, Cladomorphinae, Xenophasmina, Stephanacridini and Lanceocercata).
