# Supplementary material for: A second view on the evolution of flight in stick and leaf insects (Phasmatodea)
Source: BMC Ecol Evol. 2022 May 12;22:62. doi: 10.1186/s12862-022-02018-5 (PMC9097326; doi:10.1186/s12862-022-02018-5)
Supplement: Supplementary file 4 — Additional file 4. Supplementary discussion of phasmatodean phylogenetic relationships. [file 12862_2022_2018_MOESM4_ESM.pdf]

Additional file 4. Appendix.

Supplementary discussion of phasmatodean phylogenetic relationships

While the phasmatodean taxonomy was long considered as problematic and unsolved especially in regard to other major insect lineages [1–4], studies of recent years have illuminated much of the controversially discussed phylogenetic relationships [5–11]. Our study contributes to this progress by inferring a large-scale phylogenetic analysis of over 500 phasmatodean taxa including sequence data for nearly 200 so far unsequenced species. Despite the limited number of genes in comparison to phylogenomics studies, our results show promising similarities in the overall topology.

The New World clade Occidophasmata that was only recently revealed by phylotranscriptomic studies [6,7], was highly supported by our phylogenetic inferences, even when no constraints were set (B3 constraint; Figure S3), with *Agathemera* as sister taxon to Pseudophasmatidae [6,7,12]. Our increased taxon sampling furthermore revealed the Heteronemiinae, which have not yet been included in any phylogenomic study, as closely related to Diapheromerinae [13] and not as previously thought to Pseudophasmatidae [14–16]. By contrast, our inferences could not resolve the similarly contradicting phylogenetic position of Paraprisopodini proposed to be a clade of or closely related to Pseudophasmatidae [5,15,17,18], Diapheromerinae [19] or unrelated to these neotropical lineages [20]. Our ML inference recovered *Paraprisopus* as sister taxon to *Agathemera* (Figure S1), while their position within Pseudophasmatidae in the BI trees (Figures S2–S4) is biased due to the constraint set on Pseudophasmatidae (incl. *Paraprisopus*) for the fossil calibration. Further studies including additional members of Paraprisopodini as well as Prisopodini are needed to examine the phylogenetic relationships. That a more extensive review of the Neotropical taxa is needed was already demonstrated by Robertson et al. [5], who transferred *Otocrania* formerly assigned to Cladomorphinae to the Diapheromerinae. Bank et al. [9] found the nominal taxon *Cladomorphus* to cluster among Diapheromerinae as well, and here we additionally recover two cladomorphine members (*Cranidium* and *Hirtuleius*) within Diapheromerinae suggesting that the members of Cladomorphinae *sensu stricto* [21] in fact belong to the Diapheromerinae or Bacteriidae *sensu* Cliquennois [16]. Consequently, the remaining Cladomorphinae (Haplopodini, Hesperophasmatini, Pterinoxylini) [21] were suggested to be named Haplopodidae [16]. Furthermore, we show that also the Brazilian *Echetlus* appears to be a member of Diapheromerinae as proposed in earlier morphological studies [22,23] indicating that this taxon is not congeneric with the Southeast Asian members of *Echetlus* or the Australian Necrosciinae *Candovia* to which some of its members were later assigned [24].

Among the Old World Oriophasmata, we found much congruence with previous molecular phylogenies regarding the phylogenetic relationships of Lonchodidae (Necrosciinae + Lonchodinae) [5] and their sister taxon comprising Palophidae, Cladomorphinae (excl. *Cladomorphus*, *Cranidium* and *Hirtuleius*; see above), Pharnaciinae, Stephanacridini, *Xenophasmina* and Lanceocercata [5–7,9,11]. Within the latter, our results support the recently established Megacraniinae [22] (excl. *Apterograeffea*), albeit assuming a subordinate position as opposed to the proposed relationships by Hennemann [22]. Besides the well-supported sister groups from the Mascarene Islands (Monandropterinae *sensu* Cliquennois [16]) and from New Zealand and New Caledonia (Acanthoxylinae *sensu* Cliquennois [16]), the remaining clades of Lanceocercata (i.e., Phasmatinae, Pachymorphinae, Tropidoderinae and Xeroderinae *sensu* Cliquennois [16]) appear as highly polyphyletic [5,10,13,25,26] and are in need of formal taxonomic revision. This is particularly true with regard to the Xeroderinae, which have repeatedly been shown to be polyphyletic, and whose member *Xenophasmina* we recovered to either form the sister group to the remaining Lanceocercata (Figures S1 and S2) or to be entirely unrelated to Lanceocercata and closer related to Stephanacridini (Figures S2 and S4) [6].

The phylogenetic relationships of the remaining lineages of Oriophasmata appear rather inconsistent with the results of previous molecular analyses [5–7]. For instance the Clitumninae *sensu* Cliquennois [16] (Clitumnini + Gratidiini + Medaurini) were repeatedly shown as closely related to Pharnaciinae [6,7,12], but were here recovered in close relationship with the European Bacillinae and the African Gratidiidae *sensu* Cliquennois [16], and with the Heteropterygidae, when no constraints were set (Figures S1–S3). The low support for their relatedness, especially when Heteropterygidae are included, suggests that the topology might not be reliable. Since most of the bacilline taxa included in our inferences are represented by only one or two genes, the inferred phylogenetic position of this European group of stick insects is potentially biased, and a closer relationship to the Malagasy clade is favoured as is corroborated by phylogenomic studies and in terms of biogeography [6,7]. By contrast, the Gratidiidae (or any African taxon in general) have not yet been included in a phylogenomic study and their placement among Oriophasmata remains unclear, though Robertson et al. [5] recovered members of this lineage as close relatives to the Malagasy stick insects. In our inferences, however, the African Xylicinae are revealed as closely related to the Malagasy group. In fact, *Xylica* and an unidentified Bacillidae (possibly Xylicinae) species are highly supported as sister group to the Antongiliidae + *Spathomorpha*, while the xylicine *Bathycharax* appears unrelated to this assemblage, but is recovered as sister taxon to either the remaining Malagasy stick insects (Figures S2 and S4) or to the whole Africa/Madagascar clade (Figures S1 and S3). The African taxa are generally highly underrepresented in molecular analyses and the inclusion of several species in our study did not succeed in illuminating their evolutionary history with exception of the Palophidae, which were already previously and

repeatedly recovered as sister taxon to the Cladomorphinae [5,9,11,13]. The taxonomic shortcomings may be overcome by future work focussing on the underrepresented taxa of Africa and its associated regions. A more comprehensive taxon sampling will also be needed to elucidate the historical biogeography revolving around the colonisation of Madagascar, in particular, since our results are inconclusive about the monophyly of an African/Malagasy lineage with respect to the leaf-imitating Phylliidae. Although our results partly support the hypothesis that phylliids are closely related to European, African and Malagasy stick insects as was proposed by the phylotranscriptomic results by Simon et al. [6] in accordance with the ancestral distribution of leaf insects in Europe [27], clarification is needed to fully understand the life history of these lineages including the origin and the controversially discussed sister group of leaf insects [6,7,11].

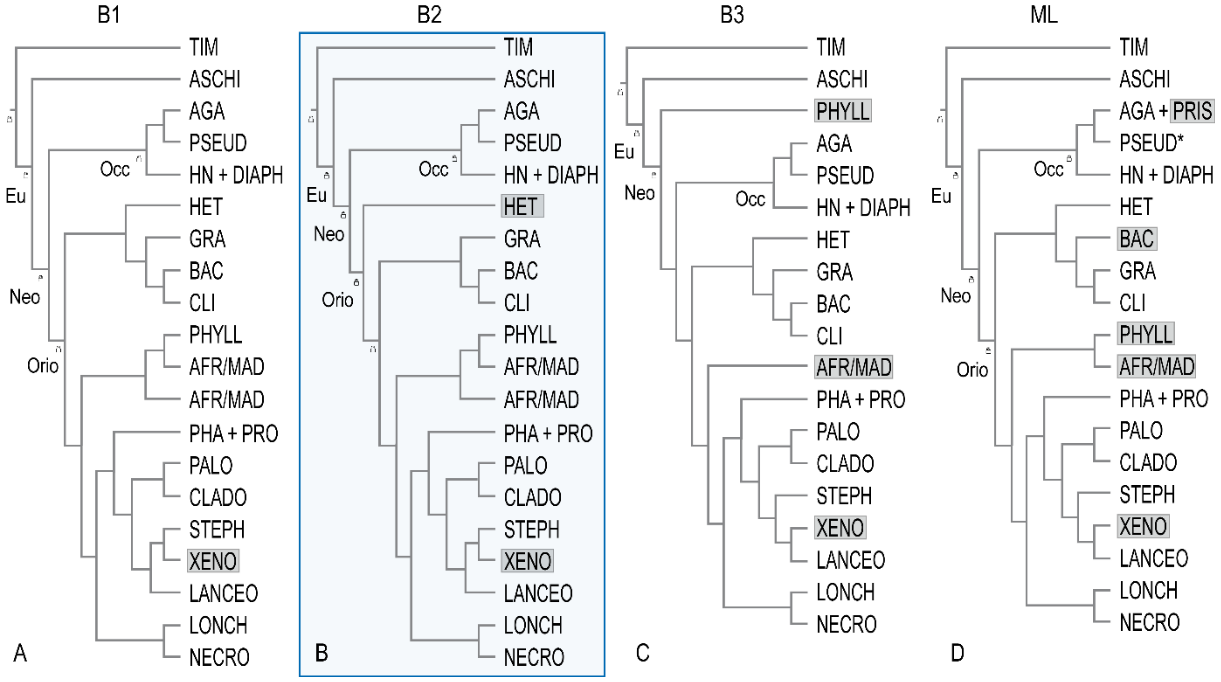

Figure A. Comparison of the four inferred phylogenies based on ML and BI methods as well as different topological constraints (B1–B3) depicted as lock symbols at the nodes (Figures S1–S4). Taxa holding a different position in comparison to the other topologies are highlighted. Blue frame indicates the topology on which subsequent analyses are based. Eu, Euphasmatodea; Neo, Neophasmatodea; Orio, Oriophasmata; Occ, Occidophasmata; TIM, Timematodea; ASCHI, Aschiphasmatidae; AGA, Agathemeridae; PSEUD, Pseudophasmatidae including *Paraprisopus*; PSEUD*, Pseudophasmatidae excluding *Paraprisopus*; HN, Heteronemiinae; DIAPH, Diapheromerinae; HET, Heteropterygidae; GRA, Gratidiidae *sensu* Cliquennois (16); BAC, Bacillinae *sensu* Cliquennois (16); CLI, Clitumninae *sensu* Cliquennois (16); AFR/MAD, African/Malagasy group including Achriopteridae, Anisacanthidae, Antongiliidae, Damasippoididae and Xylicinae *sensu* Cliquennois (16); PHYLL, Phylliidae; PHA, Pharnaciinae + *Prosentoria*; LANCEO, Lanceocercata; XENO, *Xenophasmina*; STEPH, Stephanacridiini; PALO, Palophidae; CLADO, Cladomorphinae; LONCH, Lonchodinae, NECRO, Necrosciinae.

References

1. Grimaldi D, Engel MS. Evolution of insects. Cambridge University Press, Cambridge; 2005.
2. Gullan PJ, Cranston PS. The insects - An outline of entomology. 2014.
3. GTilgner EH. Phasmida: Stick and leaf insects. In: Resh VH, Cardé RT, editors. Encyclopedia of Insects. 2nd ed. Elsevier; 2009. p. 765–66. doi: 10.1016/B978-0-12-374144-8.00203-4.
4. Engel MS, Wang B, Alqarni AS. A thorny, 'anareolate' stick-insect (Phasmatidae s.l.) in Upper Cretaceous amber from Myanmar, with remarks on diversification times among Phasmatodea. Cretac Res. 2016;63: 45–53. doi: 10.1016/j.cretres.2016.02.015.
5. Robertson JA, Bradler S, Whiting MF. Evolution of oviposition techniques in stick and leaf insects (Phasmatodea). Front Ecol Evol. 2018;6: 216. doi: 10.3389/fevo.2018.00216.
6. Simon S, Letsch H, Bank S, Buckley TR, Donath A, Liu S, et al. Old World and New World Phasmatodea: Phylogenomics resolve the evolutionary history of stick and leaf insects. Front Ecol Evol. 2019;7: 345. doi: 10.3389/fevo.2019.00345.
7. Tihelka E, Cai C, Giacomelli M, Pisani D, Donoghue PCJ. Integrated phylogenomic and fossil evidence of stick and leaf insects (Phasmatodea) reveal a Permian-Triassic co-origination with insectivores. R Soc Open Sci. 2020;7: 201689. doi: 10.1098/rsos.201689.
8. Glaw F, Hawlitschek O, Dunz A, Goldberg J, Bradler S. When giant stick insects play with colors: Molecular phylogeny of the Achriopterini and description of two new splendid species (Phasmatodea: *Achrioptera*) from Madagascar. Front Ecol Evol. 2019;7: 105. doi: 10.3389/fevo.2019.00105.
9. Bank S, Buckley TR, Büscher TH, Bresseel J, Constant J, de Haan M, et al. Reconstructing the nonadaptive radiation of an ancient lineage of ground-dwelling stick insects (Phasmatodea: Heteropterygidae). Syst Entomol. 2021;46: 487–507. doi: 10.1111/syen.12472.
10. Bradler S, Cliquennois N, Buckley TR. Single origin of the Mascarene stick insects: Ancient radiation on sunken islands? BMC Evol Biol. 2015;15: 196. doi: 10.1186/s12862-015-0478-y.
11. Bank S, Cumming RT, Li Y, Henze K, Le Tirant S, Bradler S. A tree of leaves: Phylogeny and historical biogeography of the leaf insects (Phasmatodea: Phylliidae). Commun Biol. 2021;4: 932. doi: 10.1038/s42003-021-02436-z.
12. Bradler S, Robertson JA, Whiting MF. A molecular phylogeny of Phasmatodea with emphasis on Necrosciinae, the most species-rich subfamily of stick insects. Syst Entomol. 2014;39: 205–22. doi: 10.1111/syen.12055.
13. Buckley TR, Attanayake D, Bradler S. Extreme convergence in stick insect evolution: Phylogenetic placement of the Lord Howe Island tree lobster. Proc R Soc B Biol Sci. 2009;276: 1055–62. doi: 10.1098/rspb.2008.1552.
14. Zompro O. A key to the stick-insect genera of the 'Anareolatae' of the New World, with descriptions of several new taxa (Insecta: Phasmatodea). Stud Neotrop Fauna Environ. 2004;39: 133–44. doi: 10.1080/01650520412331333783.
15. Günther K. Über die taxonomische Gliederung und geographische Verbreitung der Insektenordnung der Phasmatodea. Beiträge zur Entomol. 1953;3: 541–63. doi: 10.21248/contrib.entomol.3.5.541-563.
16. Cliquennois N. Ordre des Phasmatodea (Phasmes). In: Aberlenc H-P, editor. Les insectes du Monde. 2020. doi: 10.5962/bhl.title.122844.
17. Goldberg J, Bresseel J, Constant J, Kneubühler B, Leubner F, Michalik P, et al. Extreme convergence in egg-laying strategy across insect orders. Sci Rep. 2015;5: 7825. doi: 10.1038/srep07825.
18. Conle O V., Hennemann FH, Bellanger Y, Lelong P, Jourdan T, Valero P. Studies on neotropical Phasmatodea XX: A new genus and 16 new species from French Guiana. Zootaxa. 2020;4814: 1–136. doi: 10.11646/zootaxa.4814.1.1.
19. Forni G, Martelossi J, Valero P, Hennemann FH, Conle O V., Luchetti A, et al.

Macroevolutionary analyses provide new evidences of phasmids wings evolution as a reversible process. bioRxiv. 2020; doi: 10.1101/2020.10.14.336354.
20. Zompro O. Revision of the genera of the Areolatae, including the status of *Timema* and *Agathemera* (Insecta, Phasmatodea). Abhandlungen des Naturwissenschaftlichen Vereins in Hamburg; 2004.
21. Hennemann FH, Conle O V., Perez-Gelaber DE. Studies on Neotropical Phasmatodea XVI: Revision of Haplopodini Günther, 1953 (rev. stat.), with notes on the subfamily Cladomorphinae Bradley & Galil, 1977 and the descriptions of a new tribe, four new genera and nine new species (Phasmatodea: "Anareolatae": Phasmatidae: Cladomorphinae). Zootaxa. 2016;4128: 1–211. doi: 10.11646/zootaxa.4128.1.1.
22. Hennemann FH. Megacraniinae—The palm stick insects: A new subfamily of Old World Phasmatodea and a redefinition of Platycraninae Brunner v. Wattenwyl, 1893 (Phasmatodea: "Anareolatae"). Zootaxa. 2020;4896: 151–79. doi: 10.11646/zootaxa.4896.2.1.
23. Hennemann FH, Conle O V. Revision of Oriental Phasmatodea: The tribe Pharnaciini Günther, 1953, including the description of the world's longest insect, and a survey of the family Phasmatidae Gray, 1835 with keys to the subfamilies and tribes (Phasmatodea: "Anareolatae": Phasmatidae). Zootaxa. 2008;1906: 1–316.
24. Brock PD, Hasenpusch J. Studies on the Australian stick insects (Phasmida), including a checklist of species and bibliography. Zootaxa. 2007;1570: 1–81. doi: 10.11646/zootaxa.1570.1.1.
25. Buckley TR, Attanayake D, Nylander JAA, Bradler S. The phylogenetic placement and biogeographical origins of the New Zealand stick insects (Phasmatodea). Syst Entomol. 2010;35: 207–25. doi: 10.1111/j.1365-3113.2009.00505.x.
26. Bradler S, Buckley TR. Biodiversity of Phasmatodea. In: Foottit RG, Adler PH, editors. Insect Biodiversity: Science and Society, Volume II. 1st ed. Wiley-Blackwell, Hoboken, NJ; 2018. p. 281–313. doi: 10.1002/9781118945582.ch11.
27. Wedmann S, Bradler S, Rust J. The first fossil leaf insect: 47 million years of specialized cryptic morphology and behavior. Proc Natl Acad Sci. 2007;104: 565–9.
